# Association between colonization of the respiratory tract with *Ureaplasma species* and bronchopulmonary dysplasia in newborns with extremely low gestational age: a retrospective study

**DOI:** 10.3325/cmj.2023.64.75

**Published:** 2023-04

**Authors:** Katja Gobec, Rebeka Mukenauer, Darja Keše, Vanja Erčulj, Štefan Grosek, Tina Perme

**Affiliations:** 1 Faculty of Medicine Ljubljana, Ljubljana, Slovenia; 2 Institute of Microbiology and Immunology, Ljubljana, Slovenia; 3 Rho Sigma Research & Statistics, Ljubljana, Slovenia; 4 Department of Paediatric Surgery and Intensive Therapy, University Medical Centre Ljubljana, Ljubljana, Slovenia; 5 Department of Perinatology University Medical Centre, Ljubljana, Slovenia

## Abstract

**Aim:**

To ascertain the incidence of respiratory tract colonization in extremely low gestational age newborns (ELGANs) with *Ureaplasma parvum* and *Ureaplasma urealyticum* and determine if there is a difference in the severity of bronchopulmonary dysplasia (BPD) between ELGANs with and without *Ureaplasma* species (spp) colonization.

**Methods:**

We reviewed the medical records of ELGANs 23 0/7–27 6/7 weeks of gestation, tested for *U. parvum* and *U. urealyticum* in our Center from January 1, 2009 to December 31, 2019. *Ureaplasma* spp were identified with the Mycofast Screening Revolution assay based on liquid broth cultures or with polymerase chain reaction.

**Results:**

This study enrolled 196 preterm newborns. Fifty (25.5%) newborns had *Ureaplasma* spp respiratory tract colonization, with *U. parvum* being the predominant species. The incidence rate of respiratory tract colonization with *Ureaplasma* spp slightly increased in the studied period. The incidence rate for 2019 was 16.2 per 100 infants. BPD severity significantly correlated with *Ureaplasma* spp colonization (*P* = 0.041). After controlling for other risk factors for BPD in a regression model, preterm infants colonized with *Ureaplasma* spp had 4.32 times (95% confidence interval, CI 1.20-15.49) higher odds for developing moderate-to-severe BPD.

**Conclusions:**

*U. parvum* and *U. urealyticum* could be associated with the development of BPD in ELGANs.

Bronchopulmonary dysplasia (BPD) is a chronic lung disease affecting premature neonates and a major cause of morbidity in extremely low gestational age newborns (ELGANs; gestational age <28 weeks). The pathogenesis of BPD is complex and influenced by both prenatal and postnatal factors (1-3). Low gestational age (GA) and low birth weight, both of which reflect severe lung immaturity, are inversely correlated with the risk of developing BPD (1,2).

*Ureaplasma* species (spp), consisting of *Ureaplasma parvum* (serovars 1, 3, 6, 14) and *Ureaplasma urealyticum* (serovars 2, 4, 5, 7-13), has frequently been isolated in amniotic fluid, samples from cord blood, and respiratory tract samples from preterm infants who later developed BPD (3). *Ureaplasma* spp is a part of normal vaginal flora in 40%-80% of healthy, asymptomatic women, but its presence in the reproductive tract has been causally linked to chorioamnionitis, preterm delivery, miscarriage, and neonatal morbidity (4-9). The most common route of newborn infection is thus during passage through the colonized vaginal canal. The pathogenetic role of *Ureaplasma* spp in the development of BPD is controversial, with approaches to its detection and treatment differing greatly among European neonatal intensive care units (10). The existing studies on this issue have been difficult to interpret due to different sample sizes, inconclusive results, and differences in inclusion and diagnostic criteria for BPD (3,11).

Meta-analyses (12,13) reported higher odds for BPD development in infants colonized with *Ureaplasma* spp. Studies included in these meta-analyses used the original definition of BPD as oxygen dependence at 28 days of life (12,13). This definition has later been updated to reflect the increased survival of extremely premature infants that often need supplemental oxygen or respiratory support in the first weeks simply due to lung immaturity (14).

A growing body of evidence from both experimental models and immunological studies, due to a better understanding of virulence factors and host-pathogen interactions, supports the causal role of *Ureaplasma* spp colonization in BPD development (15-17). Respiratory tract colonization with *Ureaplasma* spp has proinflammatory and profibrogenic effects and thus contributes to the development of BPD either alone or in combination with inflammatory factors such as hyperoxia or mechanical ventilation (16). However, the impact of *Ureaplasma*-driven inflammation on neonatal morbidity has been controversial, and the clinical relevance of detecting *Ureaplasma* spp in preterm neonates remains a subject of discussion (18).

The present study was conducted in a tertiary perinatal center where the incidence of BPD has been slightly increasing in recent years despite the fact that we followed recommendations for BPD prevention, including the use of non-invasive and protective ventilation. The aim of this study was to ascertain the incidence of respiratory tract colonization in ELGANs with *U. parvum* and *U. urealyticum* and to determine whether early-life colonization with *Ureaplasma* spp is associated with the development and severity of BPD in our group of ELGANs.

## Patients and methods

### Patients

This retrospective study enrolled preterm infants with extremely low GA from 23 0/7 to 27 6/7 weeks hospitalized at the Neonatal Intensive Care and Therapy Unit (EINT), Neonatology Section, Department of Perinatology, Division of Gynecology, University Medical Centre Ljubljana in the period from January 1, 2009 to December 31, 2019, who were tested for *U. parvum* and *U. urealyticum*. We excluded all infants who died within 24 hours after birth. We collected pregnancy and perinatal data, and the data on treatment course and morbidities.

Maternal and newborn characteristics were chosen based on previous studies (19). The following prenatal maternal data were collected: age, parity, type of labor, administration of prenatal steroids and prenatal antibiotics, diagnosis of diabetes with insulin treatment, primary hypertension, chorioamnionitis and/or preeclampsia, or eclampsia.

The newborns’ data included the number of days spent in EINT, inborn or outborn delivery, death, GA, birth weight, small for GA, type of resuscitation in the delivery suite, timing of surfactant application, fraction of inspired oxygen (FiO_2_) when surfactant was administered, method of surfactant delivery (less invasive surfactant administration; minimally invasive surfactant therapy; intubation-surfactant-extubation; invasive application with longer intubation), the number of surfactant applications, the use of postnatal steroids, duration of noninvasive ventilation (continuous positive airway pressure; nasal intermittent positive pressure ventilation; high-flow nasal cannula), duration of invasive mechanical ventilation or high-frequency oscillation (HFO) ventilation, duration of iNO respiratory support, the number of days with FiO_2_>0.21, and the number of days when there was a need for respiratory support (immediately after birth or after 2-3 weeks) and vasopressor use. Additional information on the incidence of BPD was obtained from the Vermont Oxford Network.

Newborn-associated morbidities included stages of BPD, pneumothorax, necrotizing enterocolitis, early or late sepsis, systemic inflammatory response syndrome, or any other infections (cytomegalovirus infection; pneumonia or both), *U. parvum* or *U. urealyticum* infection, and the use of azithromycin.

To calculate the incidence of *Ureaplasma* spp colonization of ELGANs in EINT we also collected the number of all ELGANs treated in our institution for each year. The research was approved by the National Medical Ethics Committee of Slovenia.

### Classification of BPD

We used the BPD definition proposed by the National Heart, Lung, and Blood Institute (NHLBI) Workshop criteria (20). Three levels of disease severity are used for preterm infants with GA of less than 32 weeks: 1) mild BPD, defined as a requirement for at least 28 days of supplemental oxygen therapy and discharge or termination of supplemental oxygen therapy by 36 weeks postmenstruation age; 2) moderate BPD, defined as a requirement for at least 28 days of supplemental oxygen therapy with FiO_2_ less than 0.3 at 36 weeks postmenstruation age; and 3) severe BPD, defined as a requirement for at least 28 days of supplemental oxygen therapy with FiO_2_ 0.3 or greater at 36 weeks postmenstruation age (20). Patients were additionally divided into two groups: 1) those without BPD or with mild BPD and 2) those with moderate or severe BPD. Because the used criteria do not define the severity of BPD for newborns who die before 28 days of life, we subsequently excluded additional 5 newborns. For three newborns that died between 28 days and 36 weeks postmenstrual age we determined BPD severity based on the need for supplemental oxygen in the last days before death.

### Microbiological detection of *U. parvum* and *U. urealyticum*

Microbiological samples were obtained by tracheal aspiration (intubated infants) or nasopharyngeal swabs (non-intubated infants). In 2009 and 2010, 36 patient samples were tested with the Mycofast Screening Revolution (EliTech Diagnostic, Puteaux, France) assay based on liquid broth cultures performed according to the manufacturer’s instructions. The samples were further inoculated and cultured on a mycoplasma-selective A8 agar plate. After 24-48 hours of incubation, *Ureaplasma* colonies were observed with a stereomicroscope at 60 × magnification. In 2011, 13 (62%) samples were cultured as described, and six (29%) samples were tested with a specific multiplex real-time polymerase chain reaction (PCR, Allplex STI Essential Assay, Seegene, Seoul, South Korea). Two (9%) samples were both cultured and tested with PCR. DNA was extracted from the specimens by using the automated MagNA Pure Compact instrument and the MagNA Pure Compact Nucleic Acid Isolation Kit I with Bacteria Lysis Buffer and Proteinase K (all from Roche Diagnostics, Mannheim, Germany) pretreatment. Real-time PCR was performed on a CFX96 platform (Bio-Rad, Marnes-la-Coquette, France) according to the manufacturer´s instructions. In 2012 and afterwards, all the samples were tested with specific multiplex real-time PCR.

### Statistical analysis

Categorical variables are presented as frequencies and percentages. Continuous variables are presented as mean and standard deviation, or median and interquartile range (IQR). The association between prenatal maternal information, newborn's data and morbidities, and BPD severity was assessed with a univariate logistic regression or likelihood ratio test, as appropriate. The multiple logistic regression analysis was performed to test the association between the *Ureaplasma* spp infection and moderate to mild BPD, adjusted for other risk factors, such as GA of the child, birth weight, method of delivery room resuscitation, FiO_2_ at the time of surfactant application, the number of days with FiO_2_>0.21, and postnatal steroids administration. The significance level was set to α = 0.05. The analysis was performed with SPSS Statistics for Windows, version 27.0 (IMB Corp., Armonk, NY, USA).

## RESULTS

This study included 196 preterm newborns (34.8% of all 563 ELGANs treated in EINT in the study period). The median GA was 26 weeks (180 days; IQR 172-187 days) and the median birth weight was 725 g (IQR 620-860 g). There were 71 (37.2%) newborns diagnosed with mild BPD, 78 (40.8%) with moderate BPD, and 37 (19.4%) with severe BPD. Five (2.5%) newborns did not develop BPD and five (2.5%) died before the 28th day of life and could not be classified based on the NHLBI BPD severity criteria. Fifty newborns had *Ureaplasma* spp respiratory tract colonization (25.5%, which represents 8.9% of all ELGANs treated in EINT from 2009 till 2019). *U. parvum* was isolated in 36 (18.3%) newborns and *U. urealyticum* in 12 (6.1%). In two (1%) newborns, the *Ureaplasma* species was not determined.

### The incidence of respiratory tract colonization of ELGANs with *Ureaplasma* spp

**Figure 1:**
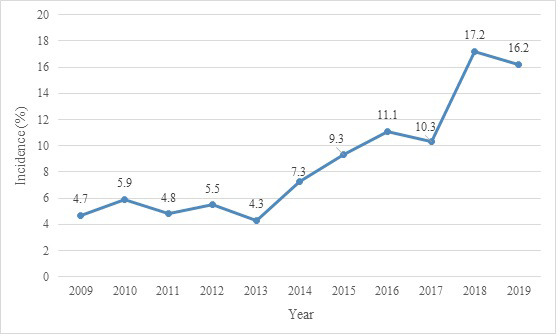
The incidence of respiratory tract colonization with *Ureaplasma* spp in extremely low gestational age newborns hospitalized at the Neonatal Intensive Care and Therapy Unit in the Maternity Hospital Ljubljana from January 1, 2009 to December 31, 2019.

**Figure 2:**
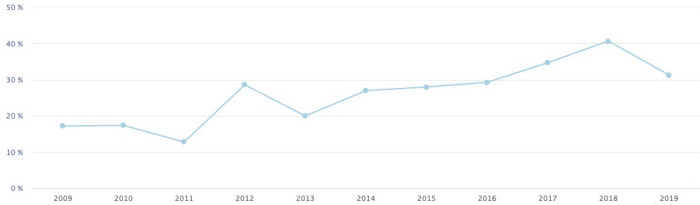
The incidence of bronchopulmonary dysplasia in extremely low gestational age newborns hospitalized at the Neonatal Intensive Care and Therapy Unit in the Maternity Hospital Ljubljana 2009 to 2019. Data were obtained from the Vermont Oxford Network.

The incidence rate of respiratory tract colonization with *Ureaplasma* spp slightly increased in the study period (Figure 1). Interestingly, BPD incidence also increased in the study period, from 17% in 2009 to 30% in 2019 (Figure 2). The average incidence rate of respiratory tract colonization with *Ureaplasma* spp was 8.8 per 100 newborns per year. The average day of testing for *Ureaplasma* spp colonization was the 23th day of life.

### Prenatal maternal characteristics and BPD

The univariate logistic regression analysis showed no significant association between prenatal maternal characteristics and the severity of BPD (Table 1).

**Table 1:** Association between prenatal maternal characteristics and bronchopulmonary dysplasia (BPD) (result of univariate logistic regression)

	BPD			
Prenatal maternal characteristics	no or mild n = 76	moderate or severe n = 113*	Odds ratio (95% confidence interval)	P
Mother's age (mean, standard deviation) (years)	31.9 (4,8)	31.6 (4.9)	0.99 (0.93; 1.05)	0.646
Number of births:				
0	49 (64.5)	75 (66.4)	1	
1	20 (26.3)	28 (24.8)	0.91 (0.46; 1.8)	0.796
2 or more	7 (9.2)	10 (8.8)	0.93 (0.33; 2.62)	0.896
Cesarean section	33 (43.4)	56 (48.7)	1.24 (0.69; 2.21)	0.475
Prenatal steroids:				
no	15 (19.7)	12 (10.6)	1	
partial	17 (22.4)	30 (26.5)	2.21 (0.84; 5.79)	0.108
full	44 (57.9)	71 (62.8)	2.02 (0.86; 4.71)	0.105
Prenatal antibiotics	50 (65.8)	70 (61.9)	0.85 (0.46; 1.55)	0.591
Insulin-dependent diabetes	2 (2.6)	2 (1.8)	0.67 (0.09; 4.84)	0.688
Primary hypertension	3 (3.9)	4 (3.5)	0.89 (0.19; 4.11)	0.884
(Pre)eclampsia	7 (9.2)	17 (15)	1.75 (0.69; 4.44)	0.242
Chorioamnionitis	14/74 (23)	25 (22.1)	0.95 (0.47; 1.92)	0.892

*we did not have the data for two mothers who gave birth outside Ljubljana Maternity Hospital, so they were excluded from this analysis.

### Newborn characteristics and BPD

The likelihood ratio test showed a significant association between BPD severity and death of newborns (*P* = 0.013) and between BPD severity and colonization with *Ureaplasma* spp (*P* = 0.041, Table 2). After controlling for other previously known risk factors for moderate-to-severe BPD, multiple logistic regression analysis showed that preterm infants colonized with *Ureaplasma* spp had 4.32 times (95% confidence interval, CI 1.20-15.49) higher odds for moderate-to-severe BPD (Table 3).

**Table 2:** Association between newborns' data and bronchopulmonary dysplasia (BPD) severity (result of univariate logistic regression)

	BPD			
Newborn characteristics	no or mild n = 76	moderate or severe n = 115	Odds ratio (95% confidence interval)	P
Number of days spent in EINT (median, IQR)	68.5 (55; 80.5)	84 (69; 97)	1.04 (1.03; 1.06)	<0.001
Outborn	5 (6.6)	14 (12.2)	1.97 (0.68; 5.71)	0.213
Death	0 (0)	6 (5.2)	-	0.013^†^
Gestational age (median; IQR) (days)	184 (174; 190)	179 (172; 184)	0.95 (0.92; 0.99)	0.008
Birth weight (median; IQR) (g)	820 (640; 960)	690 (610; 810)	1 (0.99; 1)	<0.001
Small for gestational age	9 (11.8)	20 (17.4)	1.57 (0.67; 3.65)	0.298
REA in delivery room:				
no	28 (36.8)	23 (20)	1	
mask ventilation	35 (46.1)	65 (56.5)	2.26 (1.14; 4.5)	0.020
intubation	13 (17.1)	27 (23.5)	2.53 (1.07; 5.98)	0.035
Fraction of inspired oxygen (FiO_2_; median; IQR; n) at the time of surfactant application	0.25 (0; 0.40; *72*)	0.40 (0.25; 0.55; *108*)	1.02 (1.01; 1.03)	0.002
Surfactant application:				
no	27 (40.9)	21 (19.4)	1	
less invasive	9 (13.6)	32 (29.6)	4.57 (1.8; 11.63)	0.001
invasive intubation	30 (45.5)	55 (50.9)	2.4 (1.14; 4.86)	0.02
Time (in hours after birth) of surfactant application (median; IQR; n)	1 (0; 2; *73*)	1 (0; 2; *112*)	1.01 (0.98; 1.04)	0.497
Number of surfactant applications:				
0	28 (37.8)	21 (19.1)	1	
1	37 (50)	39 (35.5)	1.41 (0.68; 2.89)	0.356
2	6 (8.1)	43 (39.1)	9.56 (3.43; 26.62)	<0.001
3 or more	3 (4.1)	7 (6.4)	3.11 (0.72; 13.48)	0.129
Number of postnatal steroids applications:				
0	52 (68.4)	29 (25.2)	1	
1	22 (28.9)	57 (49.6)	4.64 (2.38; 9.07)	<0.001
2 or more	2 (2.6)	29 (25.2)	26 (5.78; 116.89)	<0.001
Days of non-invasive ventilation (median; IQR; n)	25 (18; 35; 76)	37 (26; 48.5; *112*)	1.04 (1.02; 1.06)	<0.001
Days of MV (median; IQR)	4 (0; 11.5)	23 (13; 41)	1.07 (1.05; 1.1)	<0.001
Days of HFO (median; IQR; n)	0 (0; 0; *75*)	1 (0; 12; *113*)	1.29 (1.13; 1.46)	<0.001
Days of iNO (median; IQR; n)	0 (0; 0; *76*)	0 (0; 1; *114*)	1.27 (1.06; 1.52)	0.011
Days FiO_2_>0.21 (median; IQR)	51.5 (42; 62)	87 (74; 98)	1.11 (1.07; 1.14)	<0.001
Added oxygen:				
at the birth	54 (71.1)	97 (84.3)	1	
after 2-3 weeks	22 (28.9)	18 (15.7)	0.45 (0.22; 0.95)	0.029
Vasopressors	2 (2.6)	24 (20.9)	9.76 (2.23; 42.64)	0.002
Pneumothorax	6 (7.9)	9 (7.8)	1.03 (0.44; 2.42)	0.944
Necrotizing enterocolitis	1 (1.3)	6 (5.2)	4.13 (0.49; 34)	0.194
Early sepsis	6 (7.9)	6 (5.2)	0.64 (0.2; 2.07)	0.458
Late sepsis	8 (10.5)	24 (20.9)	2.24 (0.94; 5.3)	0.066
Systemic inflammatory response syndrome	16 (21.1)	39 (33.9)	1.92 (0.98; 3.77)	0.057
Other infections			-	0.076^†^
cytomegalovirus infection	1 (12.5)	14 (29.8)		
pneumonia	7 (87.5)	24 (51.1)		
both	0 (0)	9 (19.1)		
*Ureaplasma* spp infection	14 (18.4)	34 (29.6)	1.86 (0.92; 3.76)	0.085
*Ureaplasma* spp – bacteria:			-	0.041^†^
no	62 (81.6)	81 (70.4)		
*U. parvum*	9 (11.8)	26 (22.6)		
*U. urealyticum*	3 (3.9)	8 (6.7)		
*Ureaplasma* spp	2 (2.6)	0 (0)		
Azithromycin	13 (17.1)	30 (27.3)	1.82 (0.88; 3.77)	0.109

*Abbreviations: EINT – Neonatal Intensive Care and Therapy Unit; SD – standard deviation; IQR – interquartile range; MV – mechanical ventilation; REA – resuscitation; HFO – high-frequency oscillations, iNO – inhalatory nitrous oxide.

†Likelihood ratio test.

**Table 3:** Association between different risk factors and moderate to severe bronchopulmonary dysplasia (BPD) (results of multiple logistic regression)

Newborn's characteristics	Odds ratio (95% confidence interval)		P
Gestational age	1.1 (1.04; 1.23)	0.004	
Birth weight	1 (0.99; 1)	0.216	
Resuscitation in delivery room			
no	1		
mask ventilation	2.84 (0.84; 9.57)	0.092	
intubation	1.37 (0.29; 6.45)	0.691	
Fraction of inspired oxygen (FiO_2_) at the time of surfactant application	1 (0.98; 1.02)	0.711	
Days FiO_2_>0.21	1.13 (1.09; 1.18)	<0.001	
Postnatal steroids	2.64 (0.96; 7.26)	0.06	
*Ureaplasma* spp infection	4.32 (1.2; 15.49)	0.025	

## DISCUSSION

In this study, the development of moderate-to-severe BPD in ELGANs was significantly associated with *Ureaplasma* spp colonization. Unlike similar studies that mostly screened infants for *Ureaplasma* spp colonization soon after birth, this study enrolled infants that had their microbiological samples taken later during treatment due to a clinically suspected infection or developing BPD. Thus, 95% of the infants included in this study developed BPD according to the NHLBI diagnostic criteria. There was an important clinical and prognostic distinction between the group without or with mild BPD and the group with moderate or severe BPD, with the group without or with mild BPD having better lung function and better spirometry results (21).

Our findings agree with the findings of a meta-analysis by Schelonka et al (22), who reported a significant association between *Ureaplasma* spp colonization and BPD36 development (22). BPD36 corresponded to moderate and severe BPD according to the NHLBI criteria. However, another meta-analysis, including studies that only looked at colonization with *U. urealyticum*, did not find an association with BPD development (23). Our study did not find a difference in BPD development between *U. parvum-* and *U. urealyticum*-colonized infants; however, *U. parvum* colonization was three times as frequent (36 vs 12 infants). In the study by Glaser et al, 30/40 infants had *U. parvum*, 7/40 infants had *U. urealyticum*, and 3/40 infants had both (18).

Recent research has focused on additional risk factors for BPD that could act synergistically with *Ureaplasma* spp colonization. Inatomi et al have shown, after controlling for other risk factors, that *Ureaplasma* spp-positive infants were not at increased risk for the development of moderate-to-severe BPD. However, the association between the presence of *Ureaplasma* spp and the risk for moderate/severe BPD increased significantly in infants on mechanical ventilation ≥2 weeks (24). Similarly, the risk for BPD increased in *Ureaplasma*-colonized infants that were mechanically ventilated for five days or more, but the colonization itself was not associated with a higher risk for BPD (18).

This study found an increasing incidence of *U. parvum* and *U. urealyticum* colonization among ELGANs in Slovenia's largest tertiary perinatal center in the period 2009-2019, and a lower average incidence compared with other studies. Interestingly, we also found an increase in the incidence of BPD in ELGANs in the study period, which correlates with the increased incidence of U*reaplasma* colonization. In a study that used PCR as the method of detection, respiratory tract colonization with *Ureaplasma* spp in infants with very low birth weight (<1500 g) was 25%-48% (2). In another study, the rate in newborns younger than 26 weeks GA was 65% (25). In a more recent trial, the rate in infants younger than 29 weeks GA was 36% at one or more time points (16). In our study, the average incidence of respiratory tract colonization with *Ureaplasma* spp among ELGANs was only 8.8%.

A possible explanation for the differences between ours and other studies is a slightly different population of tested infants and a later sample collection. The mentioned studies collected microbiological samples aged <72 hours from all infants with GA younger than 26 or 29 weeks. In our study, the microbiological samples were collected later during treatment and only from infants that were clinically suspicious for developing BPD or signs of infection. The average time of testing for *Ureaplasma* spp in our institution was the 23th day after birth. In a study by Payne et al (26), the highest detection rate of *U. parvum* in aspirates from ELGANs was found 3-5 days after birth. It is worth noting, however, that three patterns of *U. urealyticum* colonization have been reported in preterm infants: persistent, early transient, and late transient. Only the persistently positive colonization pattern was associated with a significantly increased risk of developing BPD (27). It is thus possible that screening all ELGANs soon after birth would have led to a higher incidence of colonization in our institution as well. Finally, the incidence of *Ureaplasma* spp colonization among ELGANs and/or women of childbearing age could be lower in our geographical area, but we could not find any studies investigating this issue.

In our study, the frequency of testing for *Ureaplasma* spp was increasing in the period 2009-2019. In 2019, there were 15% fewer newborn ELGANs than in 2009. However, there were 4.4 times more collected microbiological samples for *Ureaplasma* spp This could in part explain the increasing incidence of colonization. Another factor contributing to the increasing incidence could be the introduction of PCR in the third year of this 11-year study, which is more sensitive than culture as a method of detection (28).

A strength of this study is the data collection from a relatively large cohort of infants over a long, 11-year period. We included infants treated in Slovenia's largest tertiary perinatal center, where 80% or more of all ELGANs receive intensive care. Our medical team used the same criteria and standards throughout the study period, which allowed us to reliably compare the year-by-year results.

This study has several limitations. First, the retrospective design prevented us from controlling the exact indications for or the timing of *Ureaplasma* spp testing and other variables. The decision to collect microbiological samples was made by the treating physician. Another limitation were the NHLBI diagnostic criteria themselves, as we could not include infants that died before the 28th day of age. Additionally, we could not use the recently proposed BPD severity diagnostic criteria that include x-ray imaging, as it was not routinely performed in our institution (29). Finally, one possible confounding factor could be the replacement of culture as the method of microbiological analysis with PCR, which is a more sensitive method that could have detected the pathogens that culture missed.

In conclusion, this study found an increasing incidence rate of *Ureaplasma* spp airway colonization in ELGANs treated in our institution in the period 2009-2019. This finding could in part be attributed to the greater proportion of tested infants and the greater sensitivity of microbiological methods. Also, BPD severity was significantly associated with the colonization with *Ureaplasma* spp. After we controlled for other risk factors for BPD in the regression model, preterm infants colonized with *Ureaplasma* spp at the average age of 23 days had 4.32-times higher odds for developing moderate or severe BPD. Screening all ELGANs soon after birth and early treatment of *Ureaplasma* spp colonization could reduce BPD incidence.

## References

[1] Thekkeveedu RT Cuevas Guaman M Shivanna B Bronchopulmonary dysplasia: a review of pathogenesis and pathophysiology. Respir Med 2017 132 170 7 10.1016/j.rmed.2017.10.014 29229093 PMC5729938

[2] Stoll BJ Hansen NI Bell EF Shankaran S Laptook AR Walsh MC Neonatal outcomes of extremely preterm infants from the NICHD Neonatal Research Network Barbara. Pediatrics 2010 126 443 56 10.1542/peds.2009-2959 20732945 PMC2982806

[3] Marie Viscardi R Hasday JD Role of Ureaplasma species in neonatal chronic lung disease: epidemiologic and experimental evidence. Pediatr Res 2009 65 84R 90R 10.1203/PDR.0b013e31819dc2f9 19190528 PMC2920621

[4] Cassell GH Waites KB Watson HL Crouse DT Harasawa R Ureaplasma urealyticum intrauterine infection: Role in prematurity and disease in newborns. Clin Microbiol Rev 1993 6 69 87 10.1128/CMR.6.1.69 8457981 PMC358267

[5] Marovt M Keše D Miljković J Matičič M Klinični pomen prisotnosti bakterij Ureaplasma parvum in Ureaplasma urealyticum v spodnjem urogenitalnem traktu žensk: Je potrebno rutinsko presejanje in zdravljenje? Zdr Vestn 2014 83 629 37

[6] Viscardi RM Ureaplasma species: Role in diseases of prematurity. Clin Perinatol 2010 37 393 409 10.1016/j.clp.2009.12.003 20569814 PMC2891804

[7] Kafetzis DA Skevaki CL Skouteri V Gavrili S Peppa K Kostalos C Maternal genital colonization with Ureaplasma urealyticum promotes preterm delivery: Association of the respiratory colonization of premature infants with chronic lung disease and increased mortality. Clin Infect Dis 2004 39 1113 22 10.1086/424505 15486833

[8] Viscardi RM Ureaplasma species: Role in neonatal morbidities and outcomes. Arch Dis Child Fetal Neonatal Ed 2014 99 87 92 10.1136/archdischild-2012-303351 23960141 PMC4239122

[9] Sprong KE Mabenge M Wright CA Govender S Ureaplasma species and preterm birth: current perspectives. Crit Rev Microbiol 2020 46 169 81 10.1080/1040841X.2020.1736986 32141797

[10] Pansieri C Pandolfini C Elie V Turner MA Kotecha S Jacqz-Aigrain E Ureaplasma, bronchopulmonary dysplasia, and azithromycin in European neonatal intensive care units: A survey. Sci Rep 2014 4 1 6 10.1038/srep04076 24518104 PMC5379254

[11] Maxwell NC Nuttall D Kotecha S Does Ureaplasma spp. cause chronic lung disease of prematurity: ask the audience? Early Hum Dev 2009 85(5)291 6 10.1016/j.earlhumdev.2008.12.002 19144476 PMC2681047

[12] Wang EEL Ohlsson A Kellner JD Association of Ureaplasma urealyticum colonization with chronic lung disease of prematurity: Results of a metaanalysis. J Pediatr 1995 127 640 4 10.1016/S0022-3476(95)70130-3 7562292

[13] Van Waarde WM Brus F Okken A Kimpen JLL Ureaplasma urealyticum colonization, prematurity and bronchopulmonary dysplasia. Eur Respir J 1997 10 886 90 10.1183/09031936.97.10040886 9150329

[14] Shennan AT Dunn MS Ohlsson A Lennox K Hoskins EM Abnormal pulmonary outcomes in premature infants: prediction from oxygen requirement in the neonatal period Pediatrics 1988 82 527 532 10.1542/peds.82.4.527 3174313

[15] Viscardi RM Kallapur SG Role of ureaplasma respiratory tract colonization in BPD pathogenesis: current concepts and update 2016 42(4)719 38 10.1016/j.clp.2015.08.003 PMC4662049 26593075

[16] Viscardi RM Terrin ML Magder LS Davis NL Dulkerian SJ Waites KB Randomised trial of azithromycin to eradicate Ureaplasma in preterm infants. Arch Dis Child Fetal Neonatal Ed 2020 105 615 22 10.1136/archdischild-2019-318122 32170033 PMC7592356

[17] Motomura K Romero R Xu Y Theis KR Galaz J Winters AD Intra-amniotic infection with ureaplasma parvum causes preterm birth and neonatal mortality that are prevented by treatment with clarithromycin. MBio 2020 11 e00797 20 10.1128/mBio.00797-20 32576673 PMC7315120

[18] Glaser K, Gradzka-Luczewska A, Szymankiewicz-Breborowicz M, Kawczynska-Leda N, Henrich B, Waaga-Gasser AM, et al. Perinatal ureaplasma exposure is associated with increased risk of late onset sepsis and imbalanced inflammation in preterm infants and may add to lung injury. Front Cell Infect Microbiol. 2019;9:68(APR):1–12. 10.3389/fcimb.2019.00068 PMC6454044 31001484

[19] Hanuna S Rus M Stucin Gantar I Erčulj V Tekavčić Pompe M Grosek Š Noninvasive ventilation for respiratory distress syndrome is a potential risk factor for retinopathy of prematurity: Single Slovenian tertiary center study. Wien Klin Wochenschr 2021 133 687 94 10.1007/s00508-021-01883-2 34081190

[20] Jobe AH Bancalari E NICHD / NHLBI / ORD workshop summary. Am J Respir Crit Care Med 2001 163 1723 9 10.1164/ajrccm.163.7.2011060 11401896

[21] Fawke J Lum S Kirkby J Hennessy E Marlow N Rowell V Lung function and respiratory symptoms at 11 years in children born extremely preterm: The EPICure study. Am J Respir Crit Care Med 2010 182 237 45 10.1164/rccm.200912-1806OC 20378729 PMC2913237

[22] Schelonka RL Katz B Waites KB Benjamin DK Critical appraisal of the role of Ureaplasma in the development of bronchopulmonary dysplasia with metaanalytic techniques. Pediatr Infect Dis J 2005 24 1033 9 10.1097/01.inf.0000190632.31565.83 16371861

[23] Zheng XD Li D Yang DH Xiang X Mei H Pu JR Association of Ureaplasma urealyticum colonization with development of bronchopulmonary dysplasia: A systemic review and meta-analysis. J Huazhong Univ Sci Technol - Med Sci 2014 34 265 9 24710943 10.1007/s11596-014-1269-1

[24] Inatomi T Oue S Ogihara T Hira S Hasegawa M Yamaoka S Antenatal exposure to Ureaplasma species exacerbates bronchopulmonary dysplasia synergistically with subsequent prolonged mechanical ventilation in preterm infants. Pediatr Res 2011 71 267 73 10.1038/pr.2011.47 22258085

[25] Sung TJ Xiao L Duffy L Waites KB Chesko KL Viscardi RM Frequency of ureaplasma serovars in respiratory secretions of preterm infants at risk for bronchopulmonary dsyplasia. Pediatr Infect Dis J 2011 30 379 83 10.1097/INF.0b013e318202ac3a 21099445 PMC3077445

[26] Payne MS Goss KCW Connett GJ Legg JP Bruce KD Chalker V A Quantitative analysis of Ureaplasma urealyticum and Ureaplasma parvum compared with host immune response in preterm neonates at risk of developing bronchopulmonary dysplasia. J Clin Microbiol 2012 50 909 14 10.1128/JCM.06625-11 22189123 PMC3295143

[27] Castro-Alcaraz S Greenberg EM Bateman DA Regan JA Patterns of colonization with Ureaplasma urealyticum during neonatal intensive care unit hospitalizations of very low birth weight infants and the development of chronic lung disease. Pediatrics 2002 110 e45 10.1542/peds.110.4.e45 12359818

[28] Waites KB Xiao L Paralanov V Viscardi RM Glass JI Molecular methods for the detection of mycoplasma and ureaplasma infections in humans: A paper from the 2011 William Beaumont Hospital symposium on molecular pathology. J Mol Diagn 2012 14 437 50 10.1016/j.jmoldx.2012.06.001 22819362 PMC3427874

[29] Higgins RD Jobe AH Koso-Thomas M Bancalari E Viscardi RM Hartert TV bronchopulmonary dysplasia: executive summary of a workshop HHS public access. J Pediatr 2018 197 300 8 10.1016/j.jpeds.2018.01.043 29551318 PMC5970962

